# Effects of undigested protein-rich ingredients on polarised small intestinal organoid monolayers

**DOI:** 10.1186/s40104-020-00443-4

**Published:** 2020-05-18

**Authors:** Soumya K. Kar, Bart van der Hee, Linda M. P. Loonen, Nico Taverne, Johanna J. Taverne-Thiele, Dirkjan Schokker, Mari A. Smits, Alfons J. M. Jansman, Jerry M. Wells

**Affiliations:** 1 Host Microbe Interactomics Group, Wageningen University & Research, De Elst 1, 6708 WD Wageningen, The Netherlands; 2 Laboratory of Microbiology, Wageningen University & Research, Stippeneng 4, 6708 WE Wageningen, The Netherlands; 3 Wageningen Livestock Research, Wageningen University & Research, De Elst 1, 6708 WD Wageningen, The Netherlands

**Keywords:** Alternative protein sources, Casein, Intestinal organoids, Organoids, Soybean meal, Spray dried plasma protein, Transcriptomics, Yellow meal worm

## Abstract

**Abstract:**

Here, we describe the use of monolayers of intestinal epithelial cells derived from intestinal organoids and transcriptomics to investigate the direct effects of dietary protein sources on epithelial function. Mechanically dissociated 3D organoids of mouse duodenum were used to generate a polarized epithelium containing all cell types found in the tissue of origin. The organoid-derived cell monolayers were exposed to 4% (w/v) of ‘undigested (non-hydrolysed)-soluble’ fraction of protein sources used as feed ingredients [soybean meal (SBM) and casein], or alternative protein sources (spray dried plasma protein, and yellow meal worm), or controls for 6 h prior to RNA isolation and transcriptomics. All protein sources altered expression of unique biological processes in the epithelial cells. Exposure of intestinal organoids to SBM downregulated expression of retinol and retinoid metabolic processes as well as cholesterol and lipid biosynthetic pathways, consistent with the reported hypotriglyceridaemic effect of soy protein *in vivo*. These findings support the use of intestinal organoids as models to evaluate complex interactions between dietary ingredients and the intestinal epithelium and highlights some unique host effects of alternative protein sources in animal feed and potentially human food.

**Graphical abstract:**

Schematic representation of the study. 3-dimensional organoids were generated from mouse duodenum (1). The organoids were subsequently dissociated into single cells (2) and grown as 2-dimensional polarised monolayers (3). Polarized monolayers of organoid cells were exposed to different protein sources [CAS, SBM, SDPP, YMW, or medium control (MC)] for 6 h (4) and further processed for imaging (5) gene expression (6), and biochemical assays (7), to investigate the effects of undigested protein sources on the duodenal epithelium. 
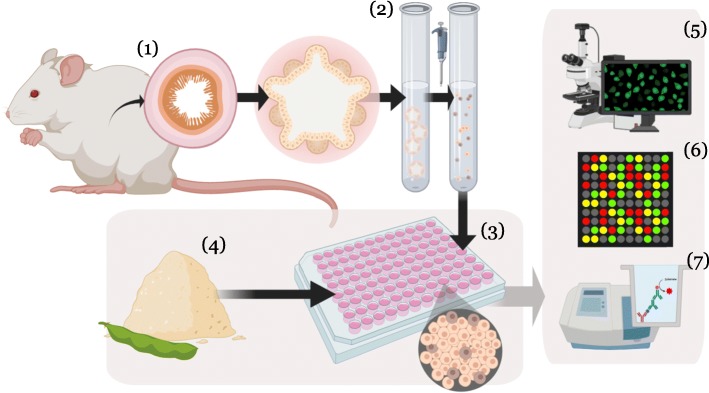

## Background

Currently, there is much interest in alternative sources of protein for animal feed, driven by the expected growth in the human population and increasing demand for animal protein. Moreover, cheaper sources of feed protein are being explored to sustain increased animal production. Alternative sources of protein include insect larvae and blood plasma. Little is known, however, about their potential non-nutritional effects on the host [1]. *In vitro* models commonly used to study intestinal responses to luminal factors include intestinal cell lines, primary cells, or tissue explants. However, cancer or transformed cell lines usually display aneuploidy, altered glycosylation patterns, and genetic mutations and deletions. Moreover, primary intestinal cells or explants are short-lived, making them less physiologically relevant for translational research [2–4].

To overcome these issues, we used three-dimensional (3D) organoid cultures from isolated crypts of the duodenum [5, 6]. Intestinal organoids can be generated within 2 weeks, maintained for > 2 years in culture or cryopreserved, and when differentiated contain all the epithelial cell lineages found at the location of origin [7, 8]. Recently, we and others have described methods for growing polarised monolayers of 3D organoid cells on semipermeable membrane supports, enabling apical exposure to luminal factors [9–11]. These two-dimensional (2D) organoid monolayers contain all the differentiated cell types found in the intestine and include many characteristics of intact, polarized epithelium, including barrier integrity and a secreted mucus layer [9, 10, 12].

Here we report on the effects of undigested (non-hydrolysed soluble fractions) of different protein sources on the transcriptome of duodenal organoids. We performed our study in duodenum organoids, as it is likely this specific location will be the primary site of undigested protein exposure *in vivo*. Soybean meal (SBM) and other protein sources being considered for animal feed such as milk casein (CAS), spray-dried plasma protein from porcine blood (SDPP), and ground freeze-dried yellow mealworm larvae (YMW) were applied to the apical surface of 2D organoid cell monolayers for 6 h after which RNA was isolated for transcriptomics using mouse gene microarrays. Potential effects of protein sources on cell viability and metabolism were measured, to control for unwanted side effects of the treatments such as apoptosis. Biochemical assays were used to validate production of triglyceride (TG) and phosphatidylcholine (PC), two pathways predicted to be activated by the different protein sources.

## Materials and methods

### 2D organoids and imaging

All protocols were approved by the animal ethics board of Wageningen University & Research, The Netherlands (2013023.f). Three nine-week-old wild type C57BL/6J female mice were purchased from Harlan Sprague Dawley Inc. (Horst, the Netherlands) and kept in the animal facility of Wageningen University & Research (12 h:12 h reversed light/dark cycle, 20 ± 2 °C). The mice were housed together in a specific pathogen-free environment with ad libitum access to a standard diet (AIN-93 M) and water. Mice were euthanized and dissected to remove the duodenum and the crypts isolated for generating organoids as previously described with minor modifications [7, 8] and grown in basal culture medium (W-ENR: DMEM/F12 medium enriched with 50 ng/mL mouse Epidermal growth factor (mEGF), 10 mmol/L Hepes (Invitrogen, the Netherlands), 1× B-27 supplement (ThermoFisher scientific, The Netherlands), and recombinant Noggin (15% v/v), WNT3A (30% v/v, Hubrecht Institute, Utrecht, Netherlands [7]), and R-Spondin (15% v/v, Stanford university, Palo Alto, USA [5]) (Method S1). To facilitate access to the apical surface of epithelial cells, we generated 2D monolayers of cells from dissociated 3D organoids. The organoids were extracted from the plate by adding 1 mL/well ice-cold DMEM F12 containing 1% v/v penicillin/streptomycin and disrupted using a p200 pipette for 40 consecutive passages and centrifuged at 250 × *g* for 5 min at 4 °C. The resulting pellet was suspended in TrypLE dissociation reagent and incubated for 10 min to receive a single-cell suspension. The cells were centrifuged at 750 × *g* for 5 min, resuspended in W-ENR at room temperature and plated at 25,000 cells per well in a 96-well culture plate, pre-coated with 0.5% Matrigel matrix as previously described [9, 10]. To confirm the presence of secretory cell lineages, the organoid monolayers were grown to confluence on 96-well plates, fixed in 1% paraformaldehyde (PFA), and subsequently visualised in histology sections with a fluorescent phalloidin conjugate (cell membranes, 1:100, A12379, Thermo-Fisher), Ki-67 (proliferating cells, 1:200, ab66155, Abcam), UEA-1 (secretory cell lineages, 1:100, FITC-conjugated, FL-1061, Vector Laboratories), and MUC2 (1:200, sc-515031, Santa Cruz Biotechnology) as previously described [9] Additional file 1.

### Protein treatments, RNA isolation, transcriptomics and biological pathway analysis in 2D organoids

Finely powdered SBM (*Glycine max*, Research diet services, The Netherlands), CAS (*Bos taurus*, Fronterra, Auckland, New Zealand), SDPP (*Sus scrofa*, Darling Ingredients, The Netherlands), and YMW (*Tenebrio molitor*, Kreca, The Netherlands) (see [13]) were added to DMEM F12 media (40 mg/mL) and homogenised using 2 cycles of 1 min with a 15 min interval on a vortex mixer . The homogenate was then allowed to settle at room temperature. Finally, the homogenate was centrifuged at 10,000 × *g* for 1 min to remove insoluble material, diluted to 400 μg/mL, and 1:10 of supernatant was added to the apical compartment of 2D organoid monolayers in W-ENR (resulting in 40 μg/mL protein; 4% w/v). After 6 h incubation at 37 °C RNA was isolated from protein-treated or medium control (DMEM-F12 only; MC) organoid monolayers for generating cDNA (Method S2). The labelled cDNA was hybridized to mouse gene 1.1 ST array (Affymetrix, Thermo Fisher Scientific, CA, USA) microarray plate and the results analysed essentially as previously described [14, 15] (Method S2). Genes differentially expressed in organoids exposed to protein sources compared to MC were identified for biological pathway analysis using GeneAnalytics (LifeMap Sciences, Inc. a subsidiary of BioTime, Inc., USA) [16]. The resulting microarray data is available in the Gene Expression Omnibus from NCBI with accession number GSE98051. Microarray results were confirmed by RT-qPCR on a selected set of differentially expressed genes (Method S3 and Table S1).

The potential effects of organoid exposure to different protein sources on cell viability and metabolism were measured using the CellTiterGlo 2.0 ATP-luminescence assay (G9241, Promega). After 6 h incubation with different protein sources, the cells were lysed using the CellTiter Glo reagent (1:1 v/v) and ATP quantified by luminescence in Spectramax M5 (Molecular Devices, Sunnyvale, CA, USA).

### Biochemical analysis of triglyceride and phosphatidylcholine

Two compounds, triglyceride (TG) and phosphatidylcholine (PC), were measured in the organoid culture supernatant for each mouse organoid line (*n* = 3 biological replicates) exposed to undigested (non-hydrolysed) protein sources using commercial detection kits (*n* = 2 per biological replicate, ab65336, and ab83377, Abcam). Samples and standards were prepared and measured according to the manufacturer’s instructions.

## Results and discussion

### Multi-cell lineage composition of 2D organoid monolayers

**Fig. 1:**
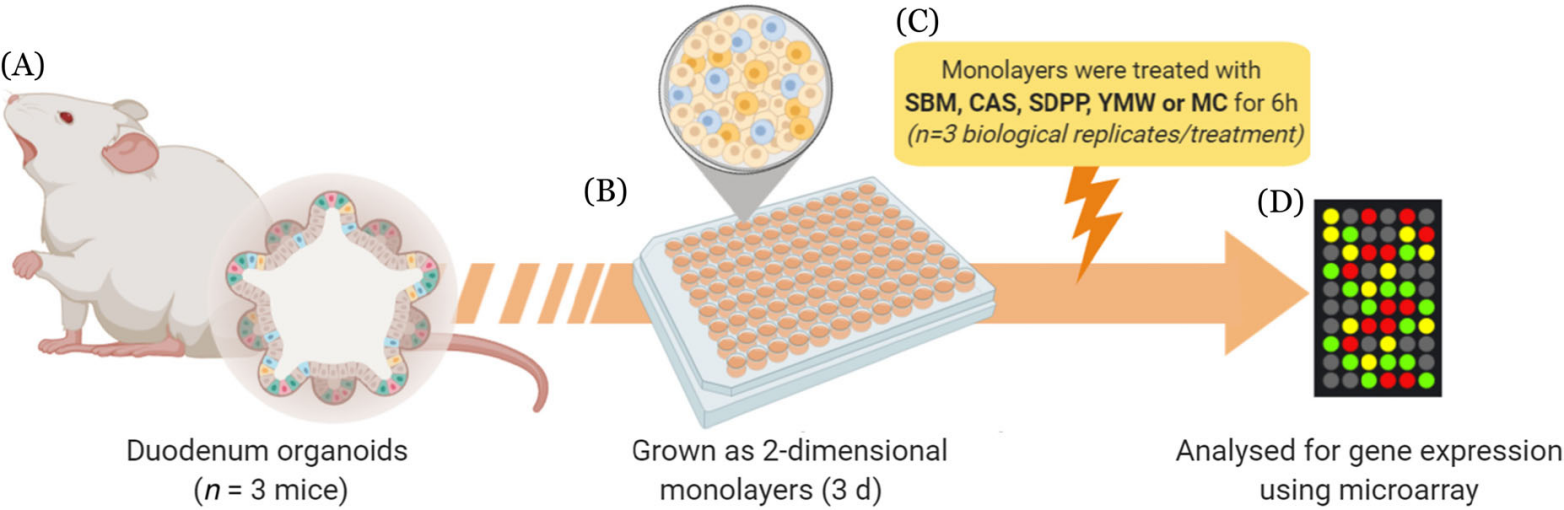
Experimental overview of the study. **a** Duodenum organoids were generated from 3 individual mice and separately cultured in Matrigel. **b** 3-dimensional organoids were single-cell dissociated by pipetting and TrypLE enzymatic digestion, plated in Matrigel coated 96-well plates, and grown until confluence for 3 d. **c** After confluence the monolayers were treated for 6 h with 4% w/v soybean meal (SBM), casein (CAS), spray-dried plasma protein (SDPP), yellow mealworm (YMW), or medium control (MC). **d** After 6 h, the monolayers were lysed and processed for gene expression profiling using Affymetrix microarrays

Two-dimensional (2D) organoid cultures from murine small intestine were generated to investigate the effects of different dietary protein sources by image analysis, transcriptomics, and biochemical assays (Fig. 1). To verify that our 2D monolayers of organoid cells contained heterotypic epithelial cell lineages as previously reported [8], we performed histological and transcriptomic analyses. Our 2D monolayers indeed contained secretory Paneth cells, and mucus-positive goblet cells as shown by immunohistochemical staining (Fig. S1), which is consistent with expression values for *Lyz* and *Muc2* transcripts in the microarray data (Fig. S2). Similarly, the genome-wide transcriptomics data revealed expression of other genes associated with specific cell lineages of intestinal epithelium including columnar base cells (CBC), and enteroendocrine cells (EEC)(Fig. S2).

### Different undigested sources of dietary protein have distinct biological effects on organoids

Organoid monolayers were exposed to different undigested protein sources (4% w/v, SBM, CAS, SDPP, and YMW) or DMEM as a MC for 6 h. Testing the effects of the treatments on cellular metabolism or survival, an ATP assay was performed. A statistically significant decline in cell ATP (< 5% compared to medium control) was observed after exposure to SBM, CAS, and YMW (Fig. S1B), which could be caused by treatment-induced changes in energy metabolism or metabolic activity. However, biologically we do not expect this to have detrimental physiological consequences on the organoid monolayers.

Principal Component Analysis (PCA) of transcriptomics data suggest separation of the treatments (95% CI) from the medium control-treated samples (PC1: 72.5% variance, Fig. S3). Analysis of the microarray data revealed that many genes were significantly (*P* < 0.05 and fold change > 1.5) differentially expressed in 2D intestinal organoids following exposure to different protein sources for 6 h (Table 1). Several of these differentially regulated genes were verified to have altered expression by RT-qPCR, showing a strong correlation (r = 0.8) to the microarray expression (Table S1). Using GeneAnalytics, the genes were matched to GO-biological processes (Table 2). Strikingly, several unique biological processes were influenced by each of the protein sources, while only one biological process was commonly regulated by all treatments (Fig. S4). For CAS, SBM, SDPP and YMW, the number of uniquely modulated GO-biological processes compared to control were 5, 8, 24 and 11, respectively.

**Table 1:** Overview of differentially expressed (*P* < 0.05) genes along with the number of related GO-biological processes in 2D organoids exposed to undigested protein sources compared to medium control (MC)

Treatment	Regulation		Differentially expressed genes ^a^	GO biological processes ^b^
CAS	Up	417	823	26
	Down	406		
SBM	Up	340	796	27
	Down	456		
SDPP	Up	262	379	40
	Down	117		
YMW	Up	391	824	20
	Down	433		

*Abbreviations*: *CAS* casein, *SBM* soybean meal, *SDPP* spray dried plasma protein, *YMW* yellow meal worm

^a^*P* < 0.05 and log(fold change) > |1.5|

^b^Analysed by GeneAnalytics; pathway analysis significance using a corrected *P*-value of < 0.05

**Table 2:** Uniquely regulated GO-biological processes of 2D organoids exposed to various protein sources. List of biological processes as analyzed in GeneAnalytics using the differentially expressed genes compared to medium control (MC)

Sl No.	Biological processes modulated in treatments compared to MC	Putative regulation of biological process
	**CAS**	
1	Glutathione metabolic process	Down
2	Notochord development	Up
3	Microtubule-based movement	Up
4	Glutathione derivative biosynthetic process	Down
5	Cellular detoxification of nitrogen compound	Down
	**SBM**	
1	Phosphatidylcholine biosynthetic process	Down
2	Triglyceride homeostasis	Down
3	Drug metabolic process	Down
4	Type I interferon signaling pathway	Up
5	Retinol metabolic process	Down
6	Hexose transport	Down
7	Lipoprotein metabolic process	Down
8	Retinoid metabolic process	Down
	**SDPP**	
1	Nucleosome assembly	Up
2	Positive regulation of apoptotic process	Up
3	Positive regulation of cell migration	Up
4	Response to wounding	Up
5	Wound healing, spreading of cells	Up
6	Negative regulation of cell cycle	Up
7	DNA replication-independent nucleosome assembly	Up
8	Cellular response to organic cyclic compound	Up
9	Angiogenesis	Up
10	Telomere organization	Up
11	Response to virus	Up
12	Cytokinesis	Up
13	DNA replication-dependent nucleosome assembly	Up
14	DNA-templated transcription, initiation	Up
15	Response to hypoxia	Up
16	Positive regulation of fever generation	Up
17	Mitotic spindle midzone assembly	Up
18	Platelet degranulation	Up
19	Positive regulation of cytokinesis	Up
20	Chromatin silencing at RDNA	Up
21	Protein stabilization	Up
22	Protein localization to kinetochore	Up
23	Omega-hydroxylase P450 pathway	Up
24	Positive regulation of protein localization to nucleus	Up
	**YMW**	
1	Cholesterol biosynthetic process	Down
2	Steroid metabolic process	Down
3	Response to drug	Down
4	Oxidation-reduction process	Up
5	Transmembrane transport	Down
6	Isoprenoid biosynthetic process	Down
7	Very long-chain fatty acid metabolic process	Down
8	CDP-choline pathway	Down
9	Long-chain fatty acid metabolic process	Down
10	Steroid hormone mediated signaling pathway	Down
11	Response to gamma radiation	Up

*Abbreviations*: *CAS* casein, *SBM* soybean meal, *SDPP* spray dried plasma protein, *YMW* yellow meal worm, *MC* medium control

^a^Analysed by GeneAnalytics; pathway analysis significance at corrected *P*-value < 0.05

^b^*P* < 0.05 and log(fold change) > |1.5|

The biological process upregulated by all protein ingredients compared to MC, was cell proliferation, indicating altered rates of cell turnover. CAS exposure to 2D organoids significantly downregulated expression of genes related to glutathione metabolic process and glutathione-derivative biosynthetic processes. Glutathione is a tripeptide, ubiquitously distributed in living cells and plays an important role in the intracellular defence mechanism against oxidative stress [17, 18]. It is known that glutathione metabolism is important for the antioxidant and detoxifying action of the intestine [19]. This suggests that CAS has detoxifying or anti-oxidative (functional) properties reducing the requirement for glutathione.

Exposure of organoids to SDPP upregulated several biological pathways associated with processes of cell migration and movement, including ‘wound healing’, ‘cytokinesis’ ‘mitotic spindle midzone assembly’, and is consistent with the upregulation of ‘DNA-templated transcription’, ‘initiation’, and ‘nucleosome assembly’. Additionally, the upregulation of angiogenesis could be related to cell apoptosis and altered expression of membrane metalloproteases and connective tissue growth factors involved in epithelial repair processes. Overall, these findings suggest increased cell turnover reflected in the upregulation of apoptosis and replication processes. The reason for this observation is unclear but may involve reconstituted serum factors from SDPP (porcine origin) and warrants further investigation.

Exposure of 2D organoids to YMW down regulated biological processes involved in lipid metabolism such as ‘steroid metabolic process’, ‘cholesterol biosynthesis process’, ‘very-long chain fatty acid metabolic process’ and ‘isoprenoid biosynthesis’. Isoprenoids are needed for biosynthesis of sterols such as cholesterol, which is vital for membrane structure. The effects of YMW are most likely to be due to the relatively high residual fat content (total fat, dry matter basis: 270 g/kg) of this protein source [13].

Soybean meal is a common protein ingredient of animal feed and down-regulated biological processes involved in triglyceride (TAG) and phosphatidylcholine (PC) biosynthesis as well as pathways associated with retinoid and retinol metabolism (Table 2). This is consistent with *in vivo* studies showing soy protein intake to be hypotriglyceridaemic in rats, having cholesterol lowering effects and reducing lipid deposition in liver [20–22].

Intestinal organoids produce and secrete chylomicrons [23], large triglyceride-rich lipoprotein particles enveloped by phospholipids, which are used for the transport of dietary fat and fat-soluble vitamins. Recently, van Rijn and colleagues showed that intestinal organoids secrete chylomicrons upon stimulation with fatty acids and measured the intracellular formation of lipid droplets [24]. To investigate whether SBM incubated organoids secreted lower amounts of these molecules we measured their concentration in the culture medium (Fig. S5). Interestingly, supernatants from SBM-treated monolayers had lower amounts of TAG and PC than the other protein treatments but were not different from MC. However, transcriptional down-regulation of these pathways was predicted from microarray data of SBM treated cells compared to the MC. This result could be explained by the presence of a minimal TAG and PC amount in the normal culture supernatant. Moreover, higher amounts of TAG and PC were found in supernatants of organoids treated with ingredients containing higher fat content than SBM (see [13]).

The hypotriglyceridaemic effect of soy protein was recently shown to be due to suppression of retinoic acid receptor expression in liver of rats fed soybean protein but not casein protein in their diet [25]. This is consistent with reports that administration of retinoids in cancer treatments are associated with hypertriglyceridemia via their interaction with retinoid receptors [26–28]. Taken together, these observations suggest that a component in SBM also negatively regulates retinoid and retinol biosynthesis as well as cholesterol and lipid biosynthetic pathways through down regulation of retinoic acid receptors in the intestinal epithelium.

In the present study we observe only part of the bio-functionality of the protein ingredients. The soluble constituents in the undigested (non-hydrolysed) soluble fraction of the protein ingredients are likely responsible for the observed effects in the present study. It can either be proteins or naturally present peptides or likely non-protein constituents that are responsible for the observed effects. However *in vivo* we can expect additional effects of the digested protein sources, in particular on metabolism. Moreover, the kinetics of protein digestion in the gastrointestinal tract differs substantially among the protein sources, particularly for SBM and SDPP [29].

## Concluding remarks

Collectively, our results indicate a direct diet-host interaction of the ‘undigested (non-hydrolysed)-soluble fraction’ of different protein sources and demonstrates that organoid monolayers are useful models to evaluate complex interaction between feed or food ingredients and the intestinal epithelium. Our transcriptome results for SBM and CAS, protein sources for which extensive studies have been performed *in vivo*, reflected those shown in animal studies. Our study suggests that a 2D intestinal organoid model can predict some effects also seen *in vivo* and might help to predict host-feed interactions.

Less studied protein sources such as SDPP and YMW also had significant effects on epithelial gene expression. For SDPP the main pathways affected indicate a possible stress response or stimulation of epithelial regeneration, for example, through increased cell turn-over [30]. Future studies can be focused on verifying the biological processes altered by SDPP and YMW in 2D intestinal organoid models, as well as fractionation of the protein sources to identify bio-molecular component(s) responsible for their effects or pre-hydrolysed fractions of other food/feed ingredients. Additionally, species-specific advances in organoid cultivation could be used to identify molecular responses of various host-species or tissue types [9, 11].

## Supplementary information

**Additional file 1: Schematic representation of the study.** 3-dimensional organoids were generated from mouse duodenum (1). The organoids were subsequently dissociated into single cells (2) and grown as 2-dimensional polarised monolayers (3). Polarized monolayers of organoid cells were exposed to different protein sources [CAS, SBM, SDPP, YMW, or medium control (MC)] for 6 h (4) and further processed for imaging (5) gene expression (6), and biochemical assays (7), to investigate the effects of undigested protein sources on the duodenal epithelium.

**Additional file 2: Materials and Methods.Method S1.** Description of crypt isolation and culture of 3D organoids. **Method S2.** RNA isolation, transcriptome and biological pathway analysis. **Method S3.** RT-qPCR.

**Additional file 3: Supplementary Tables and Figures.Table S1.** RT-qPCR primer sequences and fold-change results (average ± SEM, *n* = 3 per treatment) for microarray validation of significantly regulated genes in protein-treated organoids compared to medium control. **Fig. S1.** Organoid monolayers differentiate into polarized epithelium containing multiple cell types irrespective of treatment. (A) Staining of organoid monolayers to test for cellular morphology and proliferation (top), and secretory cell lineages, goblet (middle; MUC2) and Paneth (bottom; UEA-1) cells, when treated with various protein sources. (B) Cell ATP assay of organoids exposed to various protein sources relative to medium control (average % ± SD, *n* = 6 per treatment, * *P* < 0.05, ** *P* < 0.01). MC, medium control; SBM, soybean meal; CAS, casein; SDPP, spray dried plasma protein; YMW, yellow meal worm. **Fig. S2.** Organoid monolayers stimulated with various protein sources still maintained cell type-specific differentiation markers. Heatmap showing log_10_(expression) values of cell-specific genes in the dataset for crypt base columnar (CBC)/stem cells, label-retaining cells (+ 4 SC), niche cells, Paneth cells, goblet cells, enteroendocrine cells (EEC), absorptive enterocytes, and miscellaneous genes (MG). **Fig. S3.** PCA score plot based on genome-wide transcriptomic response measured by microarray of 2D organoids stimulated with different protein source. Colored spherical areas display 95% confidence region of respective experimental diets. Each dot represents a batch culture of organoids. MC, medium control; CAS, Casein; SBM, Soybean meal; SDPP, Spray dried plasma protein; YMW, Yellow meal worm. **Fig. S4.** Overview of non-overlapped and overlapped significant GO-biological processes modulated by protein ingredients from different sources compared to medium control, based on functional analysis results using GeneAnalytics. CAS, casein; SBM, soybean meal; SDPP, spray dried plasma protein; YMW, yellow meal worm; MC, Medium control. **Fig. S5.** Triglyceride (A) and phosphatidylcholine (B) content in supernatant of organoid monolayers stimulated for 6 h with various protein sources. Letters indicate similarity or significant differences using One-way ANOVA (concentrations given in pmol, average ± SEM, *n* = 6 monolayers per treatment derived from 3 mice, *P* < 0.05).

## Data Availability

The microarray data resulted from this study is available in the Gene Expression Omnibus of NCBI with the accession number GSE98051.

## Abbreviations

2D: Two-dimensional
3D: Three-dimensional
AIN: American Institute of Nutrition
ATP: Adenosine triphosphate
CAS: Casein
CBC: Columnar base cell
DMEM: Dulbecco’s modified Eagle medium
EDTA: Ethylenediaminetetraacetic acid
EEC: Enteroendocrine cells
FC: Fold change
GO: Gene ontology
HKG: Miscellaneous genes
IMBT: Intensity based moderated T-statistic
Lyz: Lysozyme
MC: Medium control
mEGF: Mouse epidermal growth factor
Muc2: Mucin-2
PBS: Phosphate-buffered saline solution
PC: Phosphatidylcholine
PCA: Principal component analysis
RT-qPCR: Quantitative reverse transcription PCR
SBM: Soybean meal
SC: Stem cell
SDPP: Spray dried plasma protein
TBS-T: Tris-buffered saline-tween 20
TG: Triglyceride
UEA-1: *Ulex europaeus* agglutinin 1
YMW: Yellow meal worm

## References

[1] Jahan-Mihan A, Luhovyy BL, El Khoury D, Anderson GH (2011). Dietary proteins as determinants of metabolic and physiologic functions of the gastrointestinal tract. Nutrients..

[2] Kosti I, Jain N, Aran D, Butte AJ, Sirota M. Cross-tissue analysis of gene and protein expression in normal and cancer tissues. Sci Rep. 2016;6:24799. 10.1038/srep24799. 10.1038/srep24799 PMC4855174 27142790

[3] Liu YS, Mi Y, Mueller T, Kreibich S, Williams EG, Van Drogen A (2019). Multi-omic measurements of heterogeneity in HeLa cells across laboratories. Nat Biotechnol.

[4] Randall KJ, Turton J, Foster JR (2011). Explant culture of gastrointestinal tissue: a review of methods and applications. Cell Biol Toxicol.

[5] Ootani A, Li XN, Sangiorgi E, Ho QT, Ueno H, Toda S (2009). Sustained in vitro intestinal epithelial culture within a Wnt-dependent stem cell niche. Nat Med.

[6] Sato T, Vries RG, Snippert HJ, van de Wetering M, Barker N, Stange DE (2009). Single Lgr5 stem cells build crypt-villus structures in vitro without a mesenchymal niche. Nature..

[7] Sato T, Stange DE, Ferrante M, Vries RGJ, van Es JH, van den Brink S (2011). Long-term expansion of epithelial Organoids from human Colon, adenoma, adenocarcinoma, and Barrett's epithelium. Gastroenterology..

[8] van der Hee B, Madsen O, Smidt H, Wells JM. Congruence of location-specific transcriptional programs in intestinal organoids during long-term culture. bioRxiv. 2019:600940. 10.1101/600940.

[9] van der Hee B, Loonen LMP, Taverne N, Taverne-Thiele JJ, Smidt H, Wells JM (2018). Optimized procedures for generating an enhanced, near physiological 2D culture system from porcine intestinal organoids. Stem Cell Res.

[10] VanDussen KL, Marinshaw JM, Shaikh N, Miyoshi H, Moon C, Tarr PI (2015). Development of an enhanced human gastrointestinal epithelial culture system to facilitate patient-based assays. Gut..

[11] Vogel GF, van Rijn JM, Krainer IM, Janecke AR, Posovzsky C, Cohen M, et al. Disrupted apical exocytosis of cargo vesicles causes enteropathy in FHL5 patients with Munc18–2 mutations. Jci Insight. 2017;2(14):e94564. 10.1172/jci.insight.94564. 10.1172/jci.insight.94564 PMC5518552 28724787

[12] Wang YL, DiSalvo M, Gunasekara DB, Dutton J, Proctor A, Lebhar MS (2017). Self-renewing Monolayer of Primary Colonic or Rectal Epithelial Cells. Cell Mol Gastroenter.

[13] Kar SK, Jansman AJM, Boeren S, Kruijt L, Smits MA (2016). Protein, peptide, amino acid composition, and potential functional properties of existing and novel dietary protein sources for monogastrics. J Anim Sci.

[14] Rossi O, Karczewski J, Stolte EH, Brummer RJ, van Nieuwenhoven MA, Meijerink M (2013). Vectorial secretion of interleukin-8 mediates autocrine signalling in intestinal epithelial cells via apically located CXCR1. BMC Res Notes.

[15] Sovran B, Lu P, Loonen LMP, Hugenholtz F, Belzer C, Stolte EH (2016). Identification of commensal species positively correlated with early stress responses to a compromised mucus barrier. Inflamm Bowel Dis.

[16] Ben-Ari Fuchs S, Lieder I, Stelzer G, Mazor Y, Buzhor E, Kaplan S (2016). GeneAnalytics: an integrative gene set analysis tool for next generation sequencing, RNAseq and Microarray Data. Omics.

[17] Couto N, Wood J, Barber J (2016). The role of glutathione reductase and related enzymes on cellular redox homoeostasis network. Free Radical Bio Med.

[18] Diaz-Vivancos P, de Simone A, Kiddie G, Foyer CH (2015). Glutathione - linking cell proliferation to oxidative stress. Free Radical Bio Med..

[19] Iantomasi T, Favilli F, Marraccini P, Magaldi T, Bruni P, Vincenzini MT (1997). Glutathione transport system in human small intestine epithelial cells. Bba-Biomembranes..

[20] Ascencio C, Torres N, Isoard-Acosta F, Gomez-Perez FJ, Hernandez-Pando R, Tovar AR (2004). Soy protein affects serum insulin and hepatic SREBP-1 mRNA and reduces fatty liver in rats. J Nutr.

[21] Lin YG, Meijer GW, Vermeer MA, Trautwein EA (2004). Soy protein enhances the cholesterol-lowering effect of plant sterol esters in cholesterol-fed hamsters. J Nutr.

[22] Moriyama T, Kishimoto K, Nagai K, Urade R, Ogawa T, Utsumi S (2004). Soybean beta-conglycinin diet suppresses serum triglyceride levels in normal and genetically obese mice by induction of beta-oxidation, downregulation of fatty acid synthase, and inhibition of triglyceride absorption. Biosci Biotechnol Biochem.

[23] Luchoomun J, Hussain MM (1999). Assembly and secretion of chylomicrons by differentiated Caco-2 cells - nascent triglycerides and preformed phospholipids are preferentially used for lipoprotein assembly. J Biol Chem.

[24] van Rijn JM, van Hoesel M, Middendorp S. A fluorescence-based assay for characterization and quantification of lipid droplet formation in human intestinal organoids. J Vis Exp. 2019;(152):e60150. 10.3791/60150. 10.3791/60150 31657789

[25] Torre-Villalvazo I, Tovar AR, Ramos-Barragan VE, Cerbon-Cervantes MA, Torres N (2008). Soy protein ameliorates metabolic abnormalities in liver and adipose tissue of rats fed a high fat diet. J Nutr.

[26] Radcliffe JD, Imrhan VL, Hsueh AN (1998). The use of soy protein isolate to reduce the severity of 13-cis retinoic acid-induced hypertriglyceridemia. Cancer Detect Prev.

[27] Standeven AM, Beard RL, Johnson AT, Boehm MF, Escobar M, Heyman RA (1996). Retinoid-induced hypertriglyceridemia in rats is mediated by retinoic acid receptors. Fund Appl Toxicol.

[28] Xiao CW, Mei J, Huang WX, Wood C, L'Abbe MR, Gilani GS (2007). Dietary soy protein isolate modifies hepatic retinoic acid receptor-beta proteins and inhibits their DNA binding activity in rats. J Nutr.

[29] Chen H (2017). Protein digestion kinetics in pigs and poultry.

[30] Tarnawski AS, Ahluwalia A (2012). Molecular mechanisms of epithelial regeneration and neovascularization during healing of gastric and esophageal ulcers. Curr Med Chem.

