# Supplementary material for: Effects of undigested protein-rich ingredients on polarised small intestinal organoid monolayers
Source: J Anim Sci Biotechnol. 2020 May 18;11:51. doi: 10.1186/s40104-020-00443-4 (PMC7232837; doi:10.1186/s40104-020-00443-4)
Supplement: Supplementary file 1 — Additional file 1: Schematic representation of the study. 3-dimensional organoids were generated from mouse duodenum (1). The organoids were subsequently dissociated into single cells (2) and grown as 2-dimensional polarised monolayers (3). Polarized monolayers of organoid cells were exposed to different protein sources [CAS, SBM, SDPP, YMW, or medium control (MC)] for 6 h (4) and further processed for imaging (5) gene expression (6), and biochemical assays (7), to investigate the effects of undigested protein sources on the duodenal epithelium. [file 40104_2020_443_MOESM1_ESM.docx]

**Additional file 1**


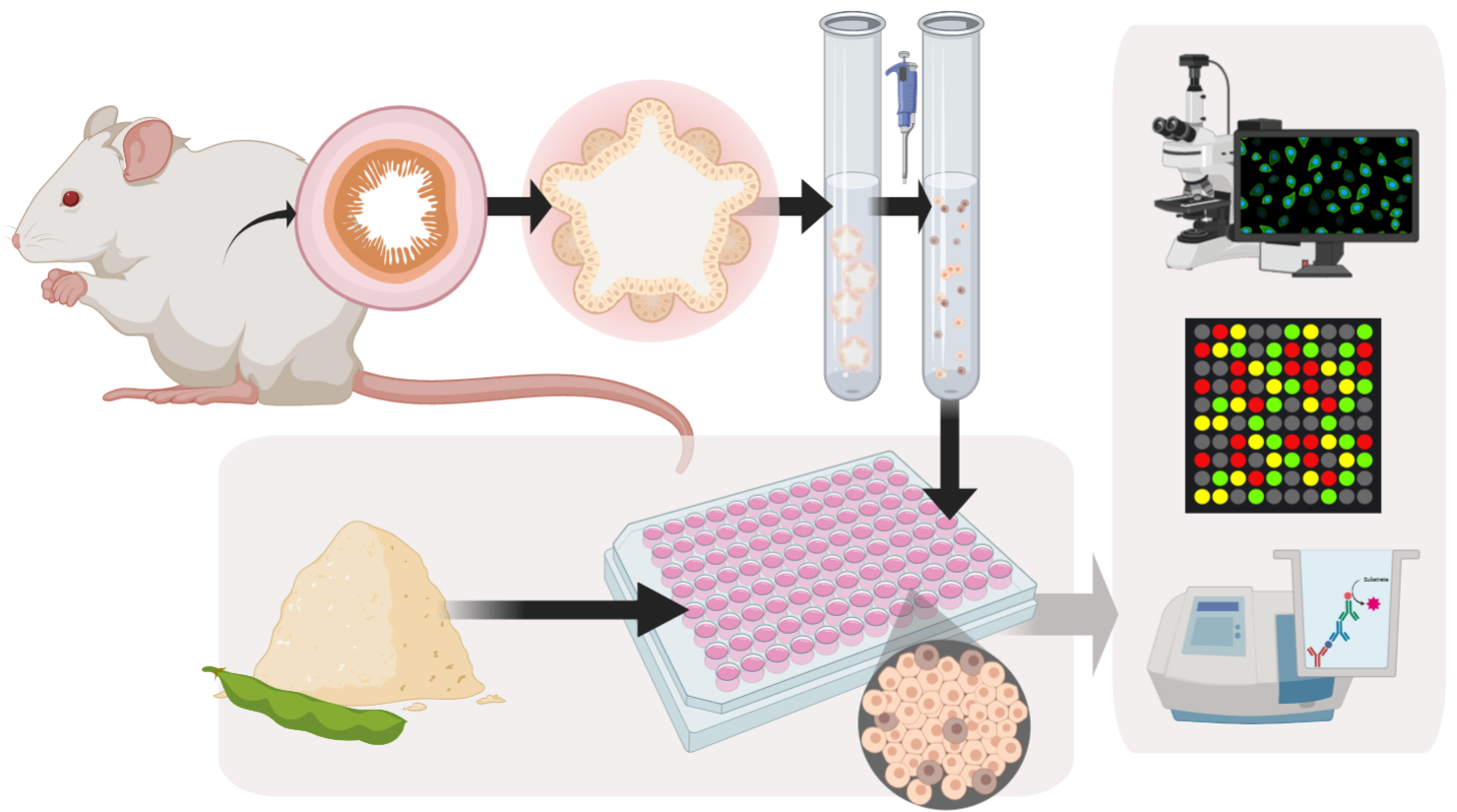


(1)

(2)

(3)

(4)

(5)

(6)

(7)

**Additional file 1. Schematic representation of the study.** 3-dimensional organoids were generated from mouse duodenum (1). The organoids were subsequently dissociated into single cells (2) and grown as 2-dimensional polarised monolayers (3). Polarized monolayers of organoid cells were exposed to different protein sources (CAS, SBM, SDPP, YMW, or medium control (MC) for 6h (4) and further processed for imaging (5) gene expression (6), and biochemical assays (7), to investigate the effects of undigested protein sources on the small intestinal epithelium.
