# Supplementary material for: Effects of undigested protein-rich ingredients on polarised small intestinal organoid monolayers
Source: J Anim Sci Biotechnol. 2020 May 18;11:51. doi: 10.1186/s40104-020-00443-4 (PMC7232837; doi:10.1186/s40104-020-00443-4)
Supplement: Supplementary file 2 — Additional file 2: Materials and Methods.Method S1. Description of crypt isolation and culture of 3D organoids. Method S2. RNA isolation, transcriptome and biological pathway analysis. Method S3. RT-qPCR. [file 40104_2020_443_MOESM2_ESM.docx]

**Additional file 2: Materials and Methods.**

**Method S1. Description of crypt isolation and culture of 3D organoids**

A two-centimeter section of the duodenum was isolated and opened longitudinally. Duodenal segments were washed in ice-cold phosphate-buffered saline solution (PBS) until the supernatant was clear. Subsequently, the tissue was incubated in PBS containing 2 mmolL ethylenediaminetetraacetic acid (EDTA) for 30 min on ice. Intestinal villi were gently removed using a glass slide, the remaining tissue was sectioned in smaller pieces, and washed with ice-cold PBS. After precipitation of the tissue fragments, PBS containing EDTA was removed and resulting fragments were thoroughly suspended in advanced Dulbecco’s modified Eagle medium (DMEM/F12, ThermoFisher scientific, the Netherlands) containing 1% *v/v* penicillin/streptomycin (PenStrep, Sigma-Aldrich, the Netherlands). Supernatant containing the crypts was filtered through a 70-µm cell strainer and centrifuged at 200 ×*g* for 3 min at 4°C. The pellet was suspended in Matrigel matrix (growth factor reduced, phenol red free, BD biosciences, the Netherlands) and plated at a density of 40-100 crypts per 50 µLin a 24-well culture plate (Corning, the Netherlands) for 3D growth of the organoids. After inverted polymerization of the matrix at 37 ⁰C with 5% CO_2_ for 20 min, 600 μL/well W-ENR was added. After seeding, the culture medium was initially replaced after 24 h and subsequently every 72 h. Organoids were sub-cultured and passaged 1:5 every 8-10 d by mechanical disruption and seeded in fresh Matrigel matrix.

**Method S2. RNA isolation, transcriptome and biological pathway analysis**

After incubation, the wells were washed with 200 μLPBS at room temperature. Total RNA was isolated using the RNeasy Mini kit (Qiagen, The Netherlands), with a 15-min on-column DNase treatment (RNAse free DNAse kit, Qiagen). RNA purity and integrity were verified using spectrophotometry (NanoDrop Technologies, USA) and Bioanalyzer (Agilent, USA). The RNA was only used to generate cDNA and perform microarray hybridization when there was no evidence of RNA degradation (RNA Integrity Number > 8). The labelling, hybridization of individual samples on Affymetrix GeneChip mouse gene 1.1 ST arrays (Affymetrix, USA), scanning, quality control and normalization of the resulting datasets was performed as described previously [1]. The resulting data is available in the Gene Expression Omnibus from NCBI with the accession number GSE98051. Differentially expressed probe sets were identified using linear models, applying moderated T-statistics that implemented empirical Bayes regularization of standard errors [2]. A Bayesian hierarchical model was used to define an intensity based moderated T-statistic (IMBT) [3]. Only genes with a fold-change (FC) of at least 1.5 (up or down) and *P*-value < 0.05 were considered significantly different. Biological interaction networks among regulated genes activated in response to protein ingredients from different sources were identified using “GeneAnalytics” (LifeMap Sciences, Inc. a subsidiary of BioTime, Inc., USA). GeneAnalytics automatically integrates gene-centric data from ~125 web sources, including genomic, transcriptomic, proteomic, genetic, clinical and functional information to identify Gene Ontology (GO) terms related to their gene sets, providing information about the molecular functions and biological roles of the genes of interest. The annotation of mice genes was performed for the subsequent functional analysis. Our GeneAnalytics analyses compared differentially regulated genes in the 2D organoids exposed to treatments compared to medium control (MC). The input was all differentially regulated genes (*P*-value < 0.05 and FC > 1.5) in the 2D organoids with/without exposure to the treatments. Here, the GO biological processes were retrieved from GeneAnalytics analysis with a high or medium score (*P*-value <0.05).

**Method S3. RT-qPCR**

The cDNA was generated from 1 microgram of total RNA by reverse transcription using a qScript cDNA synthesis kit (Quantabio, USA) according to manufacturer’s instructions and diluted 1:20. Primers were designed using Primer3 software [4-5], and purchased from Eurogentec (Oligo center, Belgium). RT-qPCR was performed using the Rotor-gene SYBR green PCR kit (Qiagen, the Netherlands) with primers specified in Table S1. Expression levels were measured in triplicate assays per sample using the Rotor-gene Q2plex real-time cycler (Qiagen). *18S* and *beta-Actin* acted as endogenous control genes and relative expression was calculated using individual amplification values, following methods described in [6]. RT-qPCR data are presented as mean ± standard error of the mean (SEM). Statistical analysis was performed by One-way analysis of variance (ANOVA) followed by Dunnet’s multiple comparisons test (Treatments vs medium control) using GraphPad prism version 5.03 (GraphPad Software, San Diego, California, USA). *P*-values < 0.05 were considered statistically significant.

**Supplementary references for Additional File 2**

1. Sovran B, Loonen LMP, Lu P, Hugenholtz F, Belzer C, Stolte EH, et al. IL-22-STAT3 Pathway Plays a Key Role in the Maintenance of Ileal Homeostasis in Mice Lacking Secreted Mucus Barrier. Inflammatory Bowel Diseases. 2015;21(3):531-42.

2. Storey J, Tibshirani R. Statistical significance for genomewide studies. Proc Natl Acad Sci USA. 2003;100.

3. Sartor MA, Tomlinson CR, Wesselkamper SC, Sivaganesan S, Leikauf GD, Medvedovic M. Intensity-based hierarchical Bayes method improves testing for differentially expressed genes in microarray experiments. Bmc Bioinformatics. 2006;7.

4. Koressaar T, Remm M. Enhancements and modifications of primer design program Primer3. Bioinformatics. 2007;23(10):1289-91.

5. Untergasser A, Nijveen H, Rao X, Bisseling T, Geurts R, Leunissen JAM. Primer3Plus, an enhanced web interface to Primer3. Nucleic Acids Research. 2007;35:W71-W4.

6. Schmittgen TD, Livak KJ. Analyzing real-time PCR data by the comparative C-T method. Nat Protoc. 2008;3(6):1101-8.
