# Supplementary material for: Effects of undigested protein-rich ingredients on polarised small intestinal organoid monolayers
Source: J Anim Sci Biotechnol. 2020 May 18;11:51. doi: 10.1186/s40104-020-00443-4 (PMC7232837; doi:10.1186/s40104-020-00443-4)
Supplement: Supplementary file 3 — Additional file 3: Supplementary Tables and Figures.Table S1. RT-qPCR primer sequences and fold-change results (average ± SEM, n = 3 per treatment) for microarray validation of significantly regulated genes in protein-treated organoids compared to medium control. Fig. S1. Organoid monolayers differentiate into polarized epithelium containing multiple cell types irrespective of treatment. (A) Staining of organoid monolayers to test for cellular morphology and proliferation (top), and secretory cell lineages, goblet (middle; MUC2) and Paneth (bottom; UEA-1) cells, when treated with various protein sources. (B) Cell ATP assay of organoids exposed to various protein sources relative to medium control (average % ± SD, n = 6 per treatment, * P < 0.05, ** P < 0.01). MC, medium control; SBM, soybean meal; CAS, casein; SDPP, spray dried plasma protein; YMW, yellow meal worm. Fig. S2. Organoid monolayers stimulated with various protein sources still maintained cell type-specific differentiation markers. Heatmap showing log10(expression) values of cell-specific genes in the dataset for crypt base columnar (CBC)/stem cells, label-retaining cells (+ 4 SC), niche cells, Paneth cells, goblet cells, enteroendocrine cells (EEC), absorptive enterocytes, and miscellaneous genes (MG). Fig. S3. PCA score plot based on genome-wide transcriptomic response measured by microarray of 2D organoids stimulated with different protein source. Colored spherical areas display 95% confidence region of respective experimental diets. Each dot represents a batch culture of organoids. MC, medium control; CAS, Casein; SBM, Soybean meal; SDPP, Spray dried plasma protein; YMW, Yellow meal worm. Fig. S4. Overview of non-overlapped and overlapped significant GO-biological processes modulated by protein ingredients from different sources compared to medium control, based on functional analysis results using GeneAnalytics. CAS, casein; SBM, soybean meal; SDPP, spray dried plasma protein; YMW, yellow meal worm; [file 40104_2020_443_MOESM3_ESM.docx]

**Additional File 3: Supplementary Tables and Figures**

**Table S1.** RT-qPCR primer sequences and fold-change results (average ± SEM, *n* = 3 per treatment) for microarray validation of significantly regulated genes in protein-treated organoids compared to medium control.

| **Gene** | **Primer forward** | **Primer reverse** | **ᵒC^*^** | **Microarray** | **qPCR** | *Correlation* |
| --- | --- | --- | --- | --- | --- | --- |
| *Stxbp1* | ATTTTCATCCTTGGGGGTGT | AAGTCGGGGTGTCTCAGGT | 60 | 4.23 | 4.57 ± 1.75 | 0.80 |
| *TNFsf13b* | TGCCTTGGAGGAGAAAGAGA | CCAGCCGAGTAGCAGGAA | 60 | 2.13 | 2.19 ± 0.83 |  |
| *Gm41* | CCTGTCCTGTTTGCTGCTCT | CTCCTTTCTCTCCTGCCTTG | 61 | 1.95 | 6.32 ± 2.33 |  |
| *Cyp1a1* | CAGAAGGTGATGGCAGAGGT | GGTAACGGAGGACAGGAATG | 60 | 1.92 | 7.00 ± 0.15 |  |
| *Olfr1162* | TGGAAAGAAATGTGAGTGTGG | TGATGGTTGAGTAGCAGAAGTC | 59 | -1.82 | -1.20 ± 0.23 |  |
| *Lims2* | GCGGATTCTGTGGTGAATTT | CTTGAACATGAGGGGCTGTT | 60 | -2.38 | -1.41 ± 0.21 |  |

^*^ Annealing temperature

**Fig. S1. Organoid monolayers differentiate into polarized epithelium containing multiple cell types irrespective of treatment.** (A) Staining of organoid monolayers to test for cellular morphology and proliferation (top), and secretory cell lineages, goblet (middle; MUC2) and Paneth (bottom; UEA-1) cells, when treated with various protein sources. (B) Cell ATP assay of organoids exposed to various protein sources relative to medium control (average % ± SD, *n* = 6 per treatment, * *P* < 0.05, ** *P* < 0.01). MC, medium control; SBM, soybean meal; CAS, casein; SDPP, spray dried plasma protein; YMW, yellow meal worm.


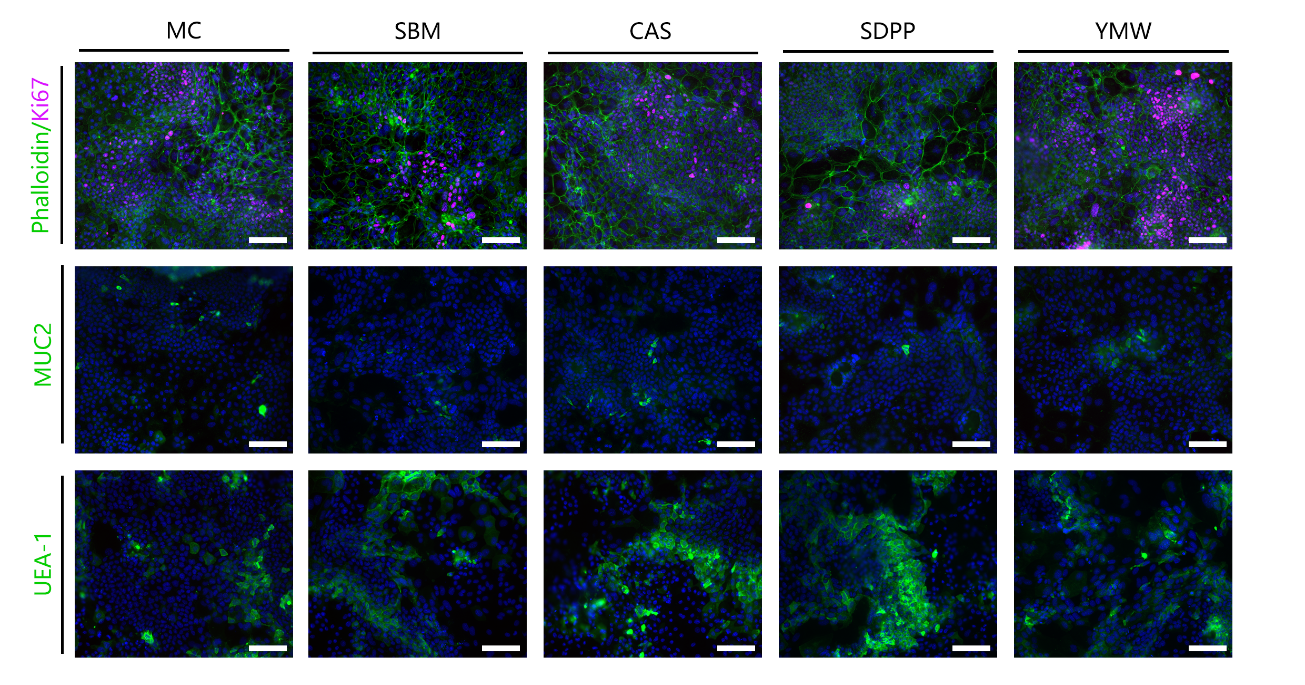

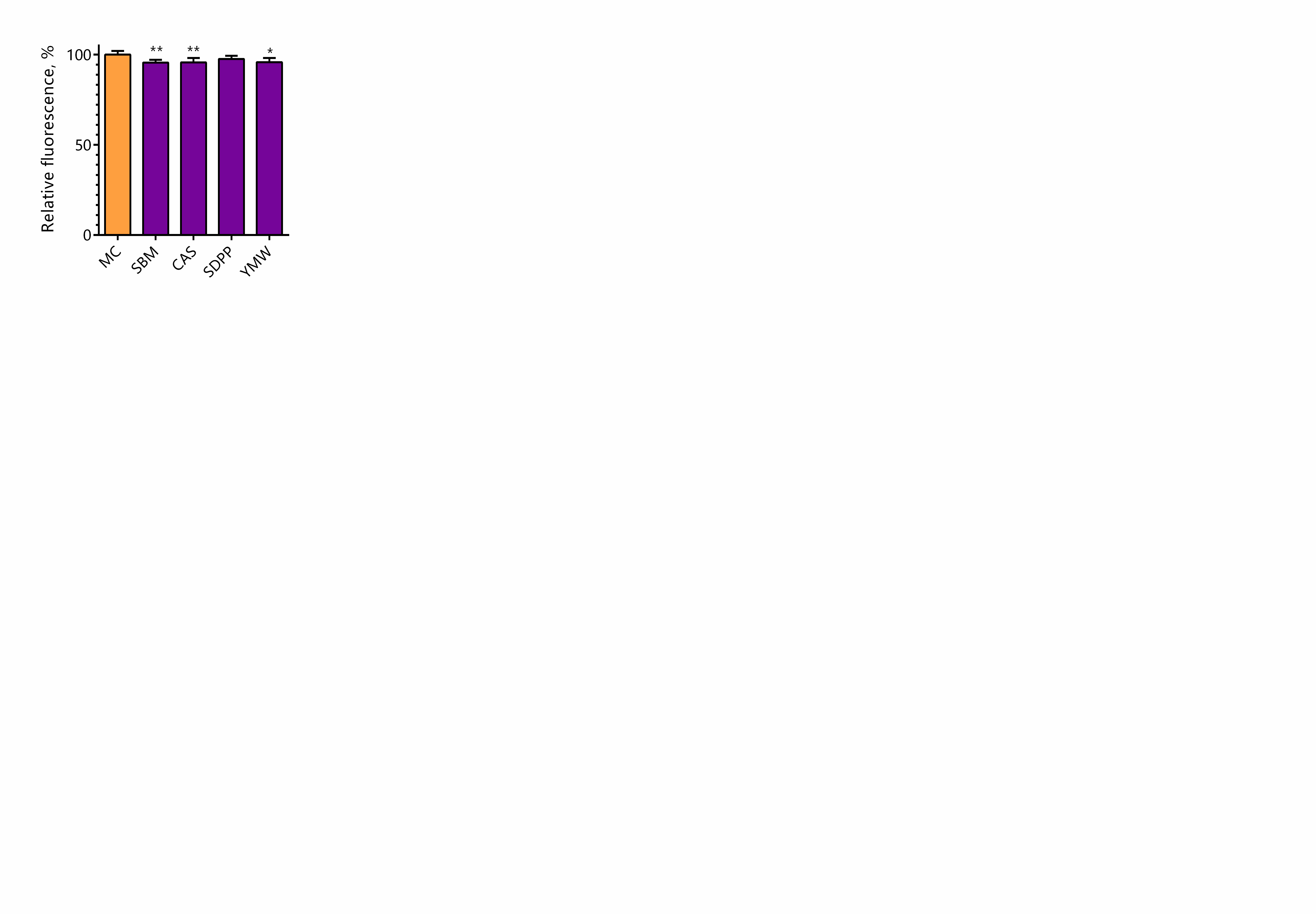


**(A)**

**(B)**


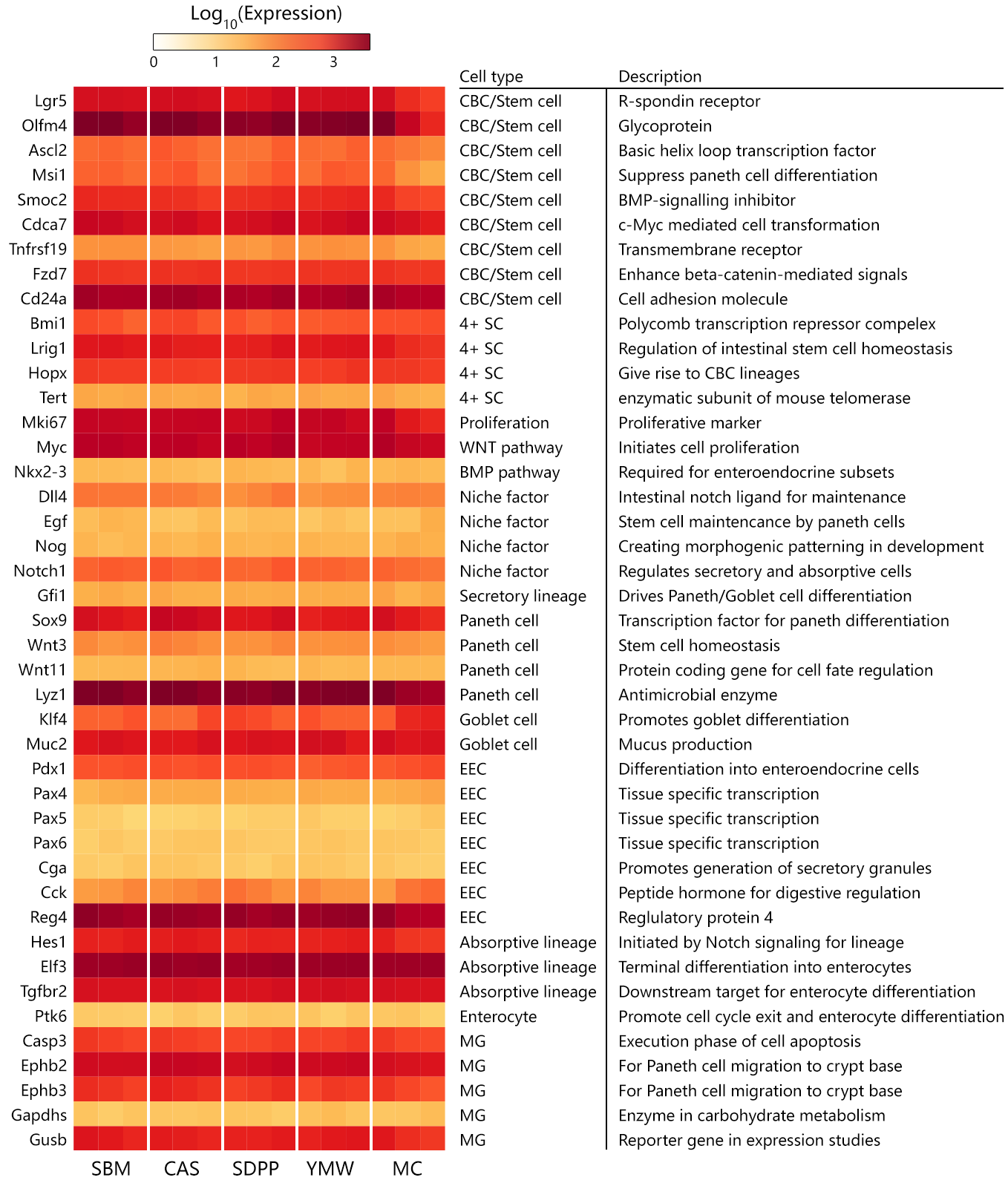
**Fig. S2.** **Organoid monolayers stimulated with various protein sources still maintained cell type-specific differentiation markers.** Heatmap showing log_10_(expression) values of cell-specific genes in the dataset for crypt base columnar (CBC)/stem cells, label-retaining cells (+4 SC), niche cells, Paneth cells, goblet cells, enteroendocrine cells (EEC), absorptive enterocytes, and miscellaneous genes (MG).

**
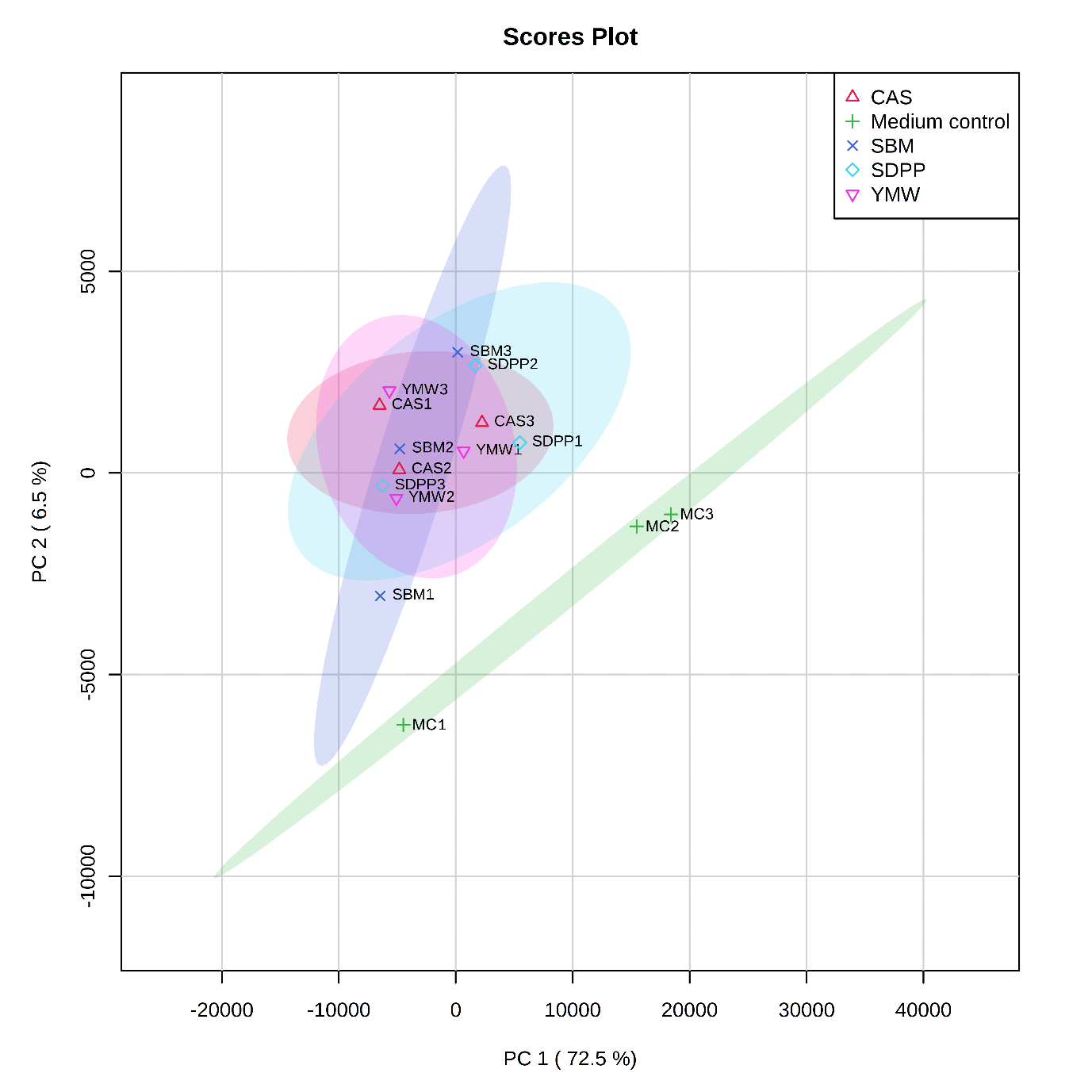
Fig. S3. PCA score plot based on genome-wide transcriptomic response measured by microarray of 2D organoids stimulated with different protein source.** Colored spherical areas display 95% confidence region of respective experimental diets. Each dot represents a batch culture of organoids. MC, medium control; CAS, Casein; SBM, Soybean meal; SDPP, Spray dried plasma protein; YMW, Yellow meal worm.

**
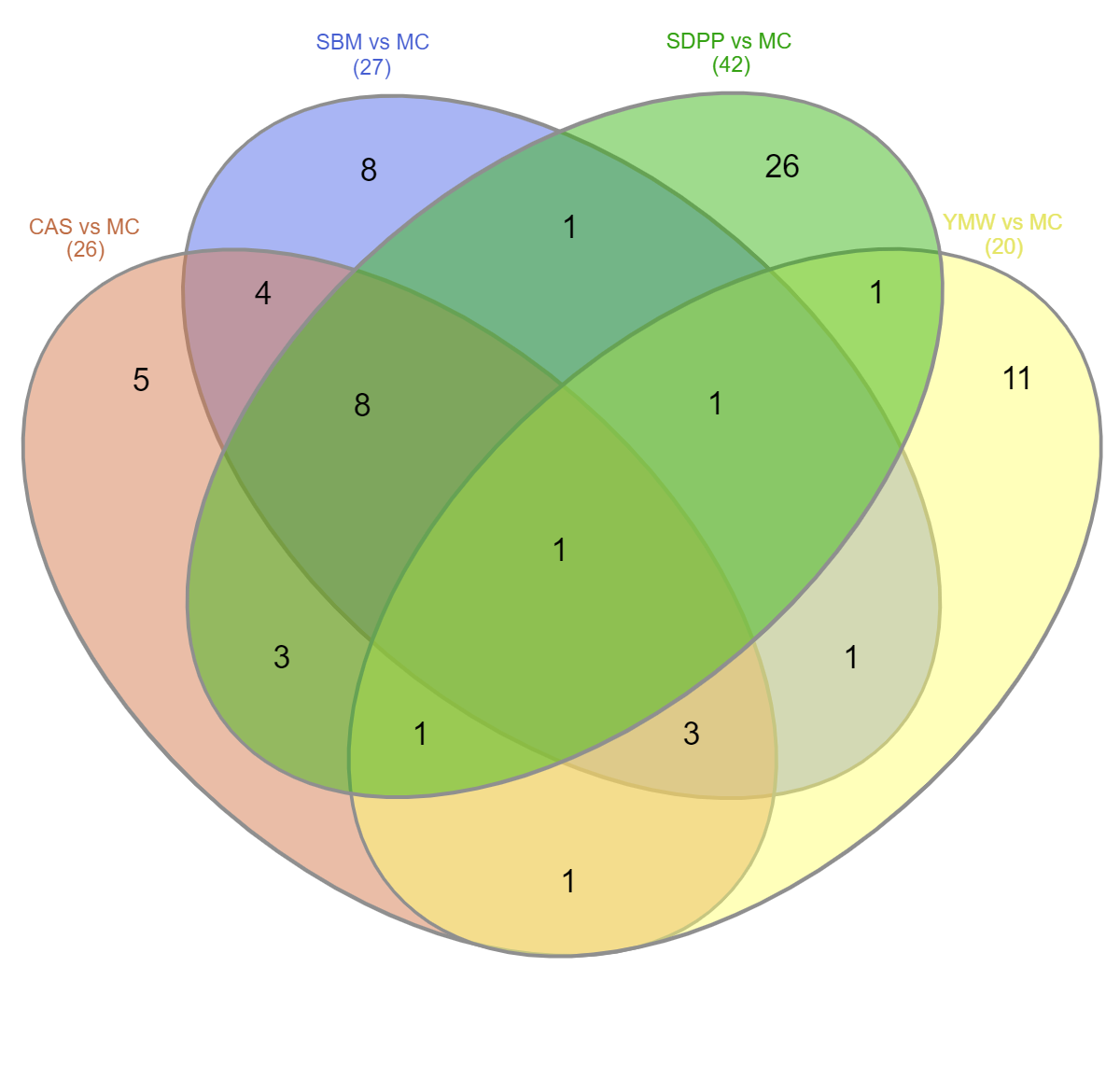
Fig. S4. Overview of non-overlapped and overlapped significant GO-biological processes modulated by protein ingredients from different sources compared to medium control, based on functional analysis results using GeneAnalytics.** CAS, casein; SBM, soybean meal; SDPP, spray dried plasma protein; YMW, yellow meal worm; MC, Medium control.

**Fig. S5. Triglyceride (A) and phosphatidylcholine (B) content in supernatant of organoid monolayers stimulated for 6 h with various protein sources.** Letters indicate similarity or significant differences using One-way ANOVA (concentrations given in pmol, average ± SEM, *n* = 6 monolayers per treatment derived from 3 mice, *P* < 0.05).


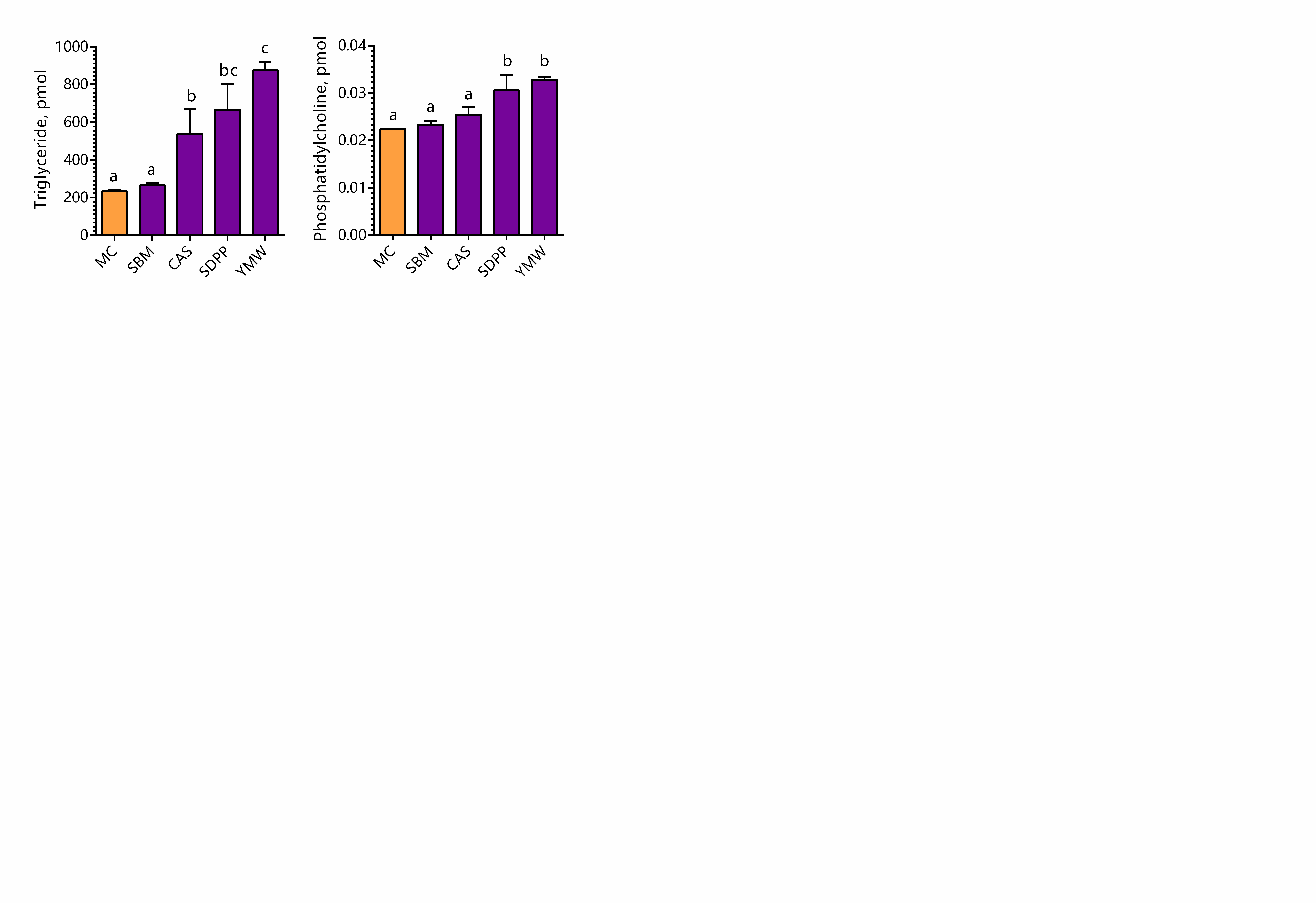


**(A)**

**(B)**
